# Numerical Study on Mie Resonances in Single GaAs Nanomembranes

**DOI:** 10.3390/nano9060856

**Published:** 2019-06-05

**Authors:** Andrés M. Raya, David Fuster, José M. Llorens

**Affiliations:** Instituto de Micro y Nanotecnología, IMN-CNM, CSIC (CEI UAM+CSIC) Isaac Newton, 8, Tres Cantos, E-28760 Madrid, Spain; andres.raya@csic.es (A.M.R.); david.fuster@csic.es (D.F.)

**Keywords:** mie resonances, GaAs nanomembranes, dielectric nanophotonics, nanoantennas

## Abstract

GaAs nanomembranes grown by selective area epitaxy are novel structures. The high refractive index of GaAs makes them good candidates for nanoantennas. We numerically studied the optical modal structure of the resonator. The nanomembrane geometry introduces a strong light-polarization dependence. The scattering is dominated by an electric dipole contribution for polarization along the nanomembrane long dimension and by a magnetic dipole contribution in the orthogonal direction. The dependence on the geometry of the resonances close to the GaAs band gap was modeled by a single coefficient. It describes the resonance shifts against up-to 40% changes in length, height, and width. We showed that the nanomembranes exhibited field enhancement, far-field directionality, and tunability with the GaAs band gap. All these elements confirm their great potential as nanoantennas.

## 1. Introduction

Antennas at the nanoscale present the ability to confine electromagnetic fields in very small volumes, far below the conventional diffraction limit. This results in a local enhancement of the fields which has been extensively exploited in plasmonics, where such nanoantennas are made out of metallic materials. The main limitation of such systems is the unavoidable presence of losses due to the excitation of conduction electrons which are characterized by high inelastic scattering rates. A similar enhancement can be achieved by dielectric resonators [1,2,3,4,5] of significant lower losses. This has triggered a great interest in this type of nanoantennas. Semiconductors are specially suited for this application as they exhibit high dielectric constants and their fabrication is well known for their relevance in electronic and optoelectronic applications. Si nanospheres [6], cylinders [7,8] disks [9], and cuboids [10] are among the best explored resonators. Also relevant are those based on direct semiconductors as they can exhibit gain. Single nanowires based on GaAs are shown to lase on their own [11] and when are embedded in plasmon nanocavities [12]. The large second-order nonlinearity coefficient of GaAs has been recently exploited in metasurfaces of nanocylinders for the second harmonic generation [13,14]. Emission has also been reported for other direct semiconductor materials, as it is the case of perovskite nanoantennas [15].

In this work, we studied a recently reported type of GaAs nanostructures exhibiting a membrane shape. They result from a careful epitaxial growth relying on a selective area pattern. By aligning rectangular openings in a SiO${}_{2}$ mask, it is possible to grow membranes on top of GaAs [16,17]. They show higher size homogeneity and, hence, narrower emission than nanowires [18]. Additionally, the nanoantenna gets its shape without the need to introduce post fabrication steps like milling or etching. These nanomembranes (NMs) offer the possibility of using them as a substrate for growing semiconductor nanostructures of large lattice mismatch [19]. A particular feature of these NMs is their asymmetric in-plane cross-section. Most of the geometries considered in the literature commonly exhibit comparable in-plane dimensions with respect to the incident light direction. This is the case for example of spheres, pyramids, and cones [20]. Our NMs are elongated in one direction, showing a strong dependence on light polarization. A similar in-plane aspect ratio is shown by a vertically standing disk, where a non-radiative anapole mode has been experimentally identified [21].

We present a numerical analysis of the Mie resonances found in GaAs NMs. Section 3.1 is devoted to describing the qualitative aspects of the nanomembrane modal structure. When the electric field is polarized parallel to the long axis of the NM, a broad electric dipole (ED) contribution to the scattering is found. In contrast, when the magnetic field is parallel to that axis, the main contribution comes from the magnetic dipole (MD) term. Due to the low symmetry of the NM, a minor contribution from the complementary dipole term is also identified. However, at certain spectral positions both contributions are of equal weight showing a dramatic reduction of the scattering in the back direction, compliant with the resonant Kerker effect [22]. From a practical point of view, it is interesting to tune the optical resonances either at the GaAs band gap, or below, in the transparency region. In this way, the nanoantenna can enhance its excitonic absorption/emission properties or those of an active nanoparticle attached to its volume for spectroscopy or biosensing [23]. A more quantitative analysis is presented in Section 3.2. We studied which geometrical parameters were more critical for tuning the NM resonances near the GaAs band-gap energy. In this Section, we propose a linear fitting rule to determine these resonances under geometrical changes of the nanomembrane. Finally, in Section 3.3, we critically review the impact a substrate would have on the light scattered by a NM.

## 2. Materials and Methods

**Figure 1:**
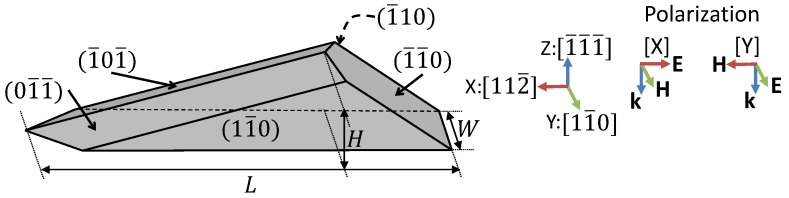
Geometrical model of the nanomembranes (NM) shape. Correspondence between axis and crytalographic directions are indicated. Field orientation of the incoming plane wave is also indicated for the polarizations *X* and *Y* considered in the text.

Figure 1 shows a reproduction of the geometrical model of the NM as derived in [24]. The preferential in-plane crystal growth direction and facets are indicated in the scheme. The geometry is defined by the length along [11$\bar{2}$] direction (*L*), the maximum height (*H*), and the thickness (*W*), as indicated in Figure 1. L=840 nm, W=120 nm, and H=198 nm are taken as starting point values for these parameters. The latter is fixed by the facets orientation and the length. *L* corresponds to the approximated average value of the GaAs band gap in nanometers between low and room temperature. The value of *W* is chosen to show a high length to width ratio.

All calculations are performed with COMSOL Multiphysics${}^{®}$ software [25]. It is used to solve Maxwell’s equations with the finite element method. The main results have been validated with SCUFF-EM, an open-source implementation of the boundary element method [26,27]. As the excitation source, we used a plane wave impinging from the top (wave vector k parallel to $-\hat{z}$) with two possible polarizations: *X* (*Y*) for electric field E parallel to that axis, i.e., parallel to the NM length (width). The NM is surrounded by air. The electromagnetic field continuity conditions are imposed at the NM boundary. The free propagation of the scattering fields away from the NM is simulated by introducing perfect matching layers as boundary conditions. The scattering ($Q_{\mathrm{scatt}}$) and absorption efficiencies ($Q_{\mathrm{abs}}$) are defined as the ratio between the scattering and absorption cross-section and the geometrical NM cross-section, i.e., its base area.

A multipole expansion of the scattering electric field in the spherical coordinate system is performed to gain a better understanding of the origin of the resonances. In a simplified notation, the scattering fields can be expanded in a complete set:

(1) $$F_{s}\left(r\right)=E_{0}\sum_{l=1}^{\infty }\sum_{m=-l}^{l}a_{E}\left(l,m\right)A_{lm}\left(r\right)+a_{M}\left(l,m\right)B_{lm}\left(r\right),$$

where $F_{s}\left(r\right)$ represents either the electric or the magnetic scattering field, $E_{0}$ is the field amplitude of the excitation source, $a_{E}\left(l,m\right)$ and $a_{M}\left(l,m\right)$ are the multipole coefficients and, $A_{lm}\left(r\right)$ and $B_{lm}\left(r\right)$ are the expansion basis functions whose definition is different for the electric and magnetic expanded field. The indices l=1,2,… and m=−l,…,l stand for the order of the multipole (dipole, quadrupole, etc.) and *m* for the *z* component of the field angular momentum. By following a projection procedure of the numerical field onto the basis elements, the coefficients can be numerically found. Full details can be found in [21,28,29]. The scattering cross-section can be easily computed [28]:

(2) $$C_{s}=\frac{\pi }{k^{2}}\sum_{l=1}^{\infty }\sum_{m=-l}^{l}\left(2l+1\right)\left[{\left|a_{E}\left(l,m\right)\right|}^{2}+{\left|a_{M}\left(l,m\right)\right|}^{2}\right],$$

where *k* is the wave vector modulus in the surrounding medium. By limiting the sum to certain values of *l*, it is possible to analyze the contribution of the different multipole terms. The field enhancement of the resonant modes is computed as the ratio of the field at its maximum point and the free-space incoming field: $E_{\max}=\max\left[E_{\mathrm{Tot}}\left(r\right)\right]/E_{0}$ for r∈Ω, being Ω the NM domain, and $E_{0}$ the amplitude of the driving-field.

The refractive index of GaAs is taken from [30,31]. The value of Im(n) is extrapolated to a constant value of 0.075 for wavelengths larger than 830 nm in order to identify the resonances in $Q_{\mathrm{abs}}$. A NM made of a material with constant refractive index (n=3.65+0.05i) is also considered in some of the calculations.

## 3. Results

### 3.1. Light Scattering by a Free Nanomembrane

NMs show excellent emission properties as reported in GaAs homoepitaxies [18]. Their good emission properties motivated us to get a better understanding of the modal structure of these NMs. In this Section, we consider only the free-standing NM case, i.e., the substrate is not included in the calculation. This analysis will describe the intrinsic optical properties of the NM. We study the impact of a glass substrate later, in Section 3.3.

**Figure 2:**
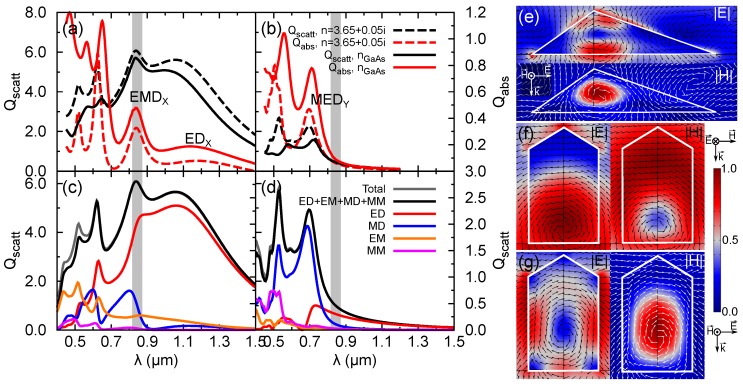
Absorption and scattering efficiencies for polarizations *X* (**a**) and *Y* (**b**). A constant refractive index n=3.65+0.05i (dashed lines) and that of GaAs (solid lines) are considered. Multipoles contribution to the scattering efficiency for constant *n* for *X* (**c**) and *Y* (**d**) polarizations. ED and MD stand for electric and magnetic dipole contributions, respectively. EM and MM stand for the sum of the contributions of higher order multipole terms. The grey shaded area shows the extension of the band-gap energy in the range of temperatures from 0 K to 300 K. |E| and |H| distribution in the plane XZ for the resonance EMD${}_{X}$ at λ = 840 nm (**e**) and in the plane YZ for the resonances ED${}_{X}$ at λ = 1175 nm (**f**) and MED${}_{Y}$ at λ = 695 nm (**g**). The vectors correspond to $\left(E_{x},E_{z}\right)$ (**e**), $\left(H_{y},H_{z}\right)$ (**f**), and $\left(E_{y},E_{z}\right)$ (**g**). The contour plots are normalized to the field maximum: 3.41 V/m, 27.8 mA/m in (**e**); 1.5 V/m, 8.76 mA/m in (**f**); 3.45 V/m, 34.36 mA/m in (**g**).

The computed $Q_{\mathrm{scatt}}$ and $Q_{\mathrm{abs}}$ of the NM for *X* and *Y* polarization are shown in Figure 2a,b, respectively. Given the novelty of the NM geometry, we want to decouple the dispersive material effects from those of the geometry. To this aim, we show the results for the refractive index of GaAs and the approximation of a constant refractive index. Excellent qualitative agreement between the results of the dispersive and non-dispersive case in this spectral region is found in Figure 2a,b. The constant *n* value case properly reproduces the fundamental features of the spectra. Therefore, the calculations in this Section rely on the non-dispersive material approximation. As mentioned in the introduction, we are interested in the spectral region close to the GaAs band gap. This spectral region is depicted in the plots with a grey shaded area. The $Q_{\mathrm{scatt}}$ for polarization *X* (Figure 2a) is dominated by a broad peak. The analysis of $Q_{\mathrm{abs}}$ permits a better estimation of the spectral features of the resonances. It is located at 1172 nm with a full width at half-maximum (FWHM) of ≈660 nm, which corresponds to a quality factor (*Q*) of ≈1.8. A peak with moderate contribution to $Q_{\mathrm{scatt}}$ but with an important increase in $Q_{\mathrm{abs}}$ can be seen exactly over the grey area. It is located at 840 nm with FWHM≈172 nm (Q≈5). We label these as ED${}_{X}$ and EMD${}_{X}$, respectively, as explained later. At wavelengths shorter than the GaAs band gap, the scattering reduces and the absorption increases. The effects of the GaAs dispersion are more pronounced here, especially due to the increase of the imaginary part of the refractive index. The features in the spectrum for the *Y* polarization are displaced towards shorter wavelengths. The scattering is significantly lower than in the case of the *X* polarization, confirming the strong dependence on the polarization anticipated in the introduction. The closest peak to the GaAs band gap is at 695 nm and we label it as MED${}_{Y}$. It is narrower (FWHM≈114 nm and Q≈6) than those identified for *X* polarization.

To understand the origin of these resonances, we have performed a multipole expansion of the scattering electric field in the spherical coordinate system. Figure 2c,d show the contribution of the dipole terms (l=1) and the added contribution of higher orders up to the fourth. The agreement between the numerical computation of $Q_{\mathrm{scatt}}$ and the approximated sum of Equation (2) is very good in the spectral range considered. We can see that the peak ED${}_{X}$ has a strong electric dipole character, which justifies the labeling. In contrast, EMD${}_{X}$ can not be considered as purely electric but contains a non-negligible contribution of the magnetic dipole term. The situation changes drastically for *Y* polarization, where MED${}_{Y}$ has a dominant magnetic character, although a small electric dipole contribution is present. The near field distribution is presented in Figure 2e,g for EMD${}_{X}$, ED${}_{X}$, and MED${}_{Y}$. For ED${}_{X}$, the norm of the electric field is characterized by a single lobe dominated by the $E_{x}$ component (Figure 2f). The magnetic field exhibits an annular distribution induced by the driving field which supports the induction of an out-of-plane electric dipole. Its circulation can be better seen from the vector field, which shows the way in which the field lines close around the lobe of the electric field. The magnetic field spreads out around the NM, because the permeability is the same in the NM and in air. This explains the better coupling to the outer modes and its low absorption. EMD${}_{X}$ in Figure 2e exhibits the complementary picture. The magnetic field presents a single lobe and the electric field shows a circulation around it. The absorption is higher as a result of a higher localization of the fields inside of the NM volume. Finally, MED${}_{Y}$ in Figure 2g also shows a single lobe for the magnetic field, whose main component is $H_{x}$. The distribution of the near-field components and the discussed dipole character of the resonances are compliant with the results reported for spheres [6] and cylinders [7]. Further details about the driving mechanism of these resonances can be found elsewhere [23,32,33].

**Figure 3:**
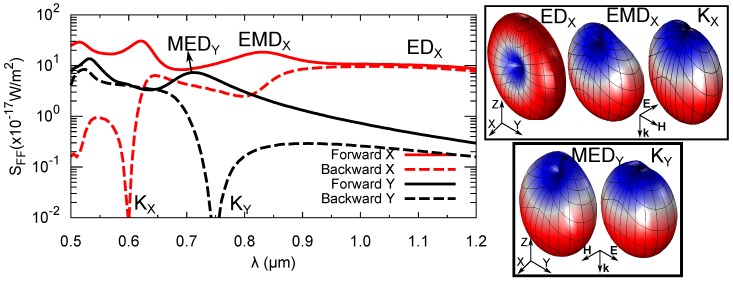
Far-field scattering flux in the backward ($-\hat{k}$) and forward ($\hat{k}$) directions for *X* and *Y* polarizations. The inset shows the far-field scattering radiation patterns at the spectral positions denoted by the labels. Same color code as in Figure 2 is used.

The far-field scattering in the forward and backward direction for both polarizations is shown in Figure 3. At long wavelengths, the forward and backward scattering are approximately equal for both polarizations. When higher-order resonances are excited, forward- and back-scattering start to depart. There is a clear correspondence between the peaks in $Q_{\mathrm{scatt}}$ of Figure 2 and the maxima in the forward-scattering, indicating that most of the scattered power is directed parallel to the excitation radiation. The ED${}_{X}$ resonance shows an almost perfect radiation pattern characteristic of an electric dipole oriented along the *X* axis. Therefore, an isotropic scattering is found in the plane normal to it. The radiation pattern of EMD${}_{X}$ still resembles the pattern of an electric dipole, although distorted by the interference with the magnetic dipole contribution. As a result, the back-scattering is reduced by 80%. For *Y* polarization, there is no resonance with a strong electric dipole contribution. However, MED${}_{Y}$ includes a non-negligible electric dipole component. This results in a radiation pattern similar to that of EMD${}_{X}$. Had it it been an isolated magnetic dipole, we should have found a pattern similar to that of ED${}_{X}$ with the same orientation, as in both cases the dipole is oriented along the *X* axis. Again, the interference between the electric and magnetic components reduces the back-scattering by 90%. At 605 nm and 774 nm there are two minima in the backward-scattering for *X* and *Y* polarization, respectively. These spectral points are labeled as K${}_{X}$ and K${}_{Y}$. The corresponding radiation patterns show a clear reduction in the upper half-plane. At these points, the dephase between the electric and magnetic dipoles results in a destructive interference. Such interference condition is known in the literature as the resonant Kerker effect [22]. It is a clear indication of the strong directionality that can be achieved by small resonators and has been reported for many geometries [9,20,34,35,36,37].

An important property of nanoantennas is the localization of electromagnetic fields. We find an enhancement of 3.0 and 3.3 (10.5 and 12.9) for the electric (magnetic) field for EMD${}_{X}$ and MED${}_{Y}$, respectively. These values are similar to the case of isolated Si nanospheres [38]. Higher enhancement values are hence expected when studying NM dimers than those found in nanospheres dimers. This issue is out of the scope of the current study and will be addressed in future work.

### 3.2. Resonance Position Dependence on NM Geometry

A quantitative analysis of the resonance position against geometrical changes of the NM is performed in the current section. The analysis is focused on the two resonances that can be tuned around the GaAs band gap, i.e., EMD${}_{X}$ and MED${}_{Y}$. The positions of the resonances are identified through the analysis of $Q_{\mathrm{abs}}$ peaks. The results of this Section include the GaAs refractive index dispersion.

**Figure 4:**
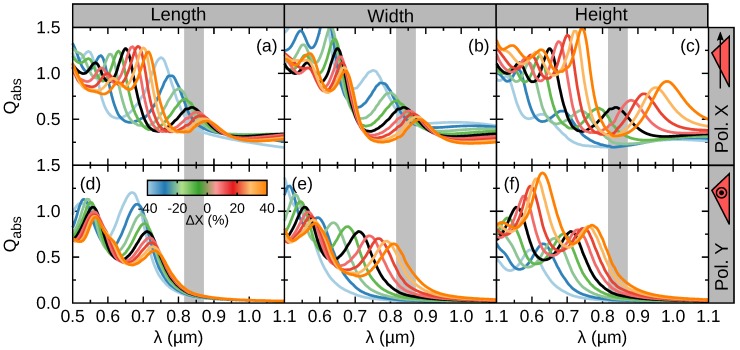
Absorption efficiency for NMs considering the relative change of the geometrical parameters length, width, and height. (**a**–**c**) Polarization vector parallel to *X* and (**d**–**f**) parallel to *Y*.

Figure 4 shows the $Q_{\mathrm{abs}}$ evolution with the change in the NM dimensions. For this purpose, we proceed by changing one parameter (*L*, *W*, or *H*) while keeping the others equal to their reference value. The line colors refer to the value of the parameter change. We quantify it as a relative change: $\Delta X=X/X_{0}-1$, where *X* represents the modified parameter and $X_{0}$ its value in the reference NM. The black line refers to ΔX=0, i.e., the spectrum shown in Figure 2. It is reproduced in all the panels as a reference. In all cases ΔX>0 results in a red-shift, while ΔX<0 in a blue-shift. The positive (negative) change in geometry results in a weaker (stronger) confinement of the fields, respectively. For polarization *X*, a broad tunability is observed by changes in the NM height. The shift covers a spectral range of half a micron. Such stronger dependence on *H* is explained by the strong vertical confinement of the electric and magnetic fields shown in Figure 2e. The width and length parameters offer smaller shifts, specially towards longer wavelengths. An intriguing result is the equal shift of EMD${}_{X}$ for equal values of ΔL and ΔW (see Figure 4a,b). However this is not the case for *Y* polarization. This effect is explained in detail later on. The MED${}_{Y}$ resonance can be shifted in a range of ∼300 nm by changing *W* and *H*. Reaching the GaAs band-gap energy would require a simultaneous change of both parameters. Again, the large shift can be understood in terms of the field confinement shown in Figure 2g.

**Figure 5:**
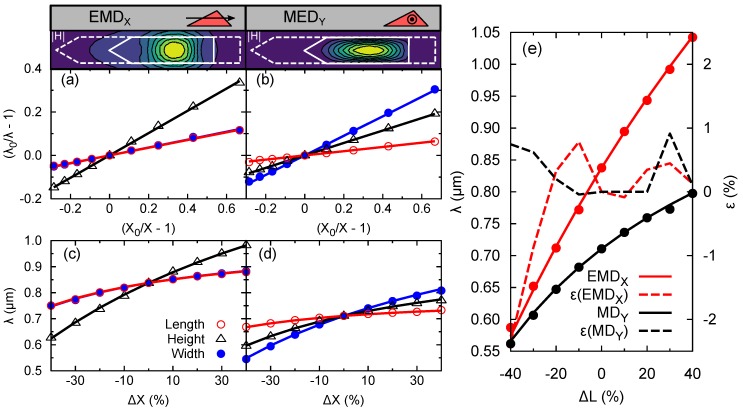
Linear dependence of the EMD${}_{X}$ (**a**) and MED${}_{Y}$ (**b**) resonances shifts as a function of the change of the geometrical parameters. Lines are the result of a linear fitting. Position of the resonances as a function of the relative parameter change (**c**,**d**). Lines are the analytical formula in Equation (3). Inset: Contour plot of |H| across the XY plane at its maximum (*z* = 85 nm). The solid line marks the NM cross-section and the dashed line the NM base cross-section. (**e**) Resonances position and estimation error for ΔL=ΔH and ΔW=0.

The complexity of NM geometry does not allow the prediction of the peaks position from the Fabry–Perot interference condition [39]. Instead, we will take a close look at the position of these resonances in order to get an empirical rule to make such a prediction. The dependence of the position as a function of the parameter change is explicitly shown in Figure 5. It shows a linear dependence when represented as a relative change of wavelength and geometry, i.e., as the ratio $\left(\lambda_{0}/\lambda -1\right)$ versus $\left(X_{0}/X-1\right)$ (Figure 5a,b). The value of $\lambda_{0}$ is the spectral position in the reference NM (Figure 2). A linear fitting provides a straightforward prediction of the absorption peak position for geometrical changes of the NM. The values of the fitting parameter ($a_{X}$) are included in Table 1. This parameter can be interpreted as the deformation potential used in electronic structure calculations to determine the bands edges position as a function of the strain of the crystal unit cell [40]. In the analogy, the role of the strain is played here by ΔX. Note that, in our context, ΔX only describes a change in geometry, not an elastic deformation. For practical purposes, it is more useful to show the dependence of λ on ΔX (see Figure 5c,d). With only one fitting constant, we can reproduce very accurately the non-linear evolution of the resonance position with ΔX:

(3) $$\lambda =\lambda_{0}/\left[1-a_{X}\Delta X/\left(1+\Delta X\right)\right].$$

The coincidence of the resonance shift for ΔL and ΔW for EMD${}_{X}$ is now more evident. In contrast, this behavior is not observed for MED${}_{Y}$. Its origin is at the different confinement regimes of the fields in each case. In the inset of Figure 5, we show a cross-section of |H| at the resonant wavelength. It is found that the confinement along the *X* and *Y* axis is very similar for EMD${}_{X}$. The contour lines span over similar distances and hence a deformation of *L* or *W* results in a similar spectral shift. The contour lines of MED${}_{Y}$ are notably elongated along the *X* axis, i.e., the field is less strongly confined along this direction than along the orthogonal one. This results in a stronger shift for a deformation of *W* with respect to a deformation of *L*, as shown in Figure 5b.

In actual fabricated NMs, it is possible to find changes in more than one geometrical parameter. Indeed, to preserve the crystal planes, the NM requires a constant ratio L/H. The NMs are grown by a selected area epitaxy, meaning that they grow at openings in a dielectric thin slab. The substrate is exposed to the growth chamber only at such openings [16,17,24]. Hence, the openings will fix the *L* and *W* parameters. To describe this situation, we can use the above derived rule to determine the position of EMD${}_{X}$ and MED${}_{Y}$:

(4) $$\lambda =\lambda_{0}{\left(\prod_{X=L,W,H}1-\frac{a_{X}\Delta X}{1+\Delta X}\right)}^{-1}.$$

The accuracy of Equation (4) is illustrated in Figure 5e. There, we show the evolution of the resonance position for different values of *L* keeping L/H fixed (ΔL=ΔH and ΔW=0). The symbols indicate the numerical results and the lines are the result of applying Equation (4) with the coefficients of Table 1. We have also included the results of the relative error between the prediction and the numerical results. As can be seen, the error is always smaller than 2% for polarization *X* and even smaller for polarization *Y*. Our simple analytical expression allows for fine tuning the main resonances found in the GaAs NM for geometrical changes, which can be as large as 40%.

### 3.3. Substrate Effect on the Scattering Properties

In previous sections, we characterized the modal properties of a free NM. NMs are currently grown by epitaxial methods on top of GaAs substrates, i.e., the same material comprising the NM. In this situation, the scattering capabilities of the NM will be notably hindered, as a result of the reduction in the contrast refractive index between the NM and the substrate. To obtain almost free standing NMs, a possibility would be to grow the nanoantennas on top of an AlGaAs sacrificial layer, which could later be removed by a selective chemical etching. Such a procedure has been routinely employed to fabricate photonic crystals in suspended slabs [41] and also nanoantennas [13]. Another possibility would be to spin a resin over the NM and remove the sacrificial layer in a second step. This would allow the transfer of structures to other substrates, like glass, for example. A similar procedure has been reported to transfer GaAs nanowires to flexible substrates [42]. In this section, we will show the impact of a glass substrate ($n_{\mathrm{subs}}=1.5$) on the scattering properties of the NM.

**Figure 6:**
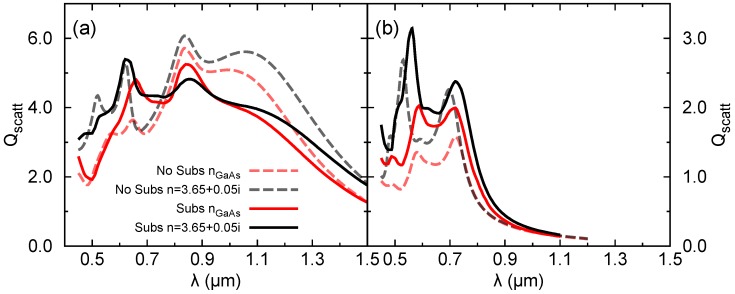
Impact of a glass substrate on the scattering properties of a NM for *X* (**a**) and *Y* (**b**) polarizations. Same geometry and conditions as in Figure 2 are used.

Figure 6 shows $Q_{\mathrm{scatt}}$ for the same NM as in Figure 2 with the inclusion of a substrate underneath. The substrate reduces the total scattered power for the long wavelength resonances (ED${}_{X}$ and EMD${}_{X}$). In contrast, for resonances at shorter wavelengths, the scattering power might even increase. Such an increase is more pronounced for the *Y* polarization. An increase with $n_{\mathrm{subs}}$ in the scattering of a magnetic dipole resonance is also reported in [7]. It is attributed to the increase of the local density of states at the position of the particle. In general, we can conclude that the substrate effects are more important for those resonances characterized by a lower *Q*-factor. For ED${}_{X}$, the fields spread farther from the resonator and are therefore more sensitive to the substrate presence. The resonances EMD${}_{X}$ and MED${}_{Y}$ are only slightly perturbed, which make the conclusions of the previous Sections still valid.

## 4. Conclusions

In summary, we studied the modal structure of recently reported GaAs NMs. The large in-plane asymmetry of the NM resulted in the strong dependence of the scattering and absorption on light polarization. Moreover, the main dipolar contribution on the scattering swapped from electric to magnetic for two orthogonal polarizations. An accurate description of the resonance position is proposed by using a single fitting coefficient. Interestingly, we identified the concurrence of electric and magnetic dipole contributions. This offers a great potential for controlling the directionality of the far-field. On the near-field side, a field enhancement similar to other all-dielectric nanoantennas has been found. Our results will help future studies on these NMs, so as to exploit their potential for light steering and sensing applications. 

## Figures and Tables

**Table 1:** Fitting coefficient $a_{X}$ and error. $a_{X}$ values are scaled by a factor $\times 10^{3}$.

Polarization	*X*	*Y*
$\lambda_{0}$ (nm)	838	711
$a_{L}$	177 ± 3	97 ± 1
$a_{W}$	182 ± 3	444 ± 6
$a_{H}$	514 ± 5	287 ± 3
